# STructured lifestyle education for people WIth SchizophrEnia (STEPWISE): mixed methods process evaluation of a group-based lifestyle education programme to support weight loss in people with schizophrenia

**DOI:** 10.1186/s12888-019-2282-5

**Published:** 2019-11-13

**Authors:** Rebecca Gossage-Worrall, Daniel Hind, Katharine D. Barnard-Kelly, David Shiers, Angela Etherington, Lizzie Swaby, Richard I. G. Holt, on behalf of The STEPWISE Research Group, Richard I. G. Holt, Katharine Barnard-Kelly, Rebecca Gossage-Worrall, Mike Bradburn, Daniel Hind, David Saxon, Lizzie Swaby, Paul French, John Pendlebury, Stephen Wright, Glenn Waller, Paul McCrone, Tiyi Morris, Charlotte Edwardson, Kamlesh Khunti, Melanie Davies, Marian Carey, Yvonne Doherty, Alison Northern, Janette Barnett, Richard Laugharne, Chris Dickens, Kathryn Greenwood, Fiona Gaughran, Sridevi Kalidindi, Shanaya Rathod, Najma Siddiqi, Angela Etherington, David Shiers

**Affiliations:** 1 Clinical Trials Research Unit, School of Health and Related Research, The University of Sheffield, Regent Court, 30 Regent Street, Sheffield, S1 4DA UK; 2 Faculty of Health & Social Science, Bournemouth University, Poole, Dorset, UK; 3 Honorary Research Consultant, Psychosis Research Unit, Greater Manchester Mental Health NHS Foundation Trust and Honorary Reader in Early Psychosis, School of Health Sciences, Division of Psychology and Mental Health, University of Manchester, Manchester, UK; 4 Patient Representative, Independent Service User Consultant, Manchester, UK; 5 Human Development and Health, Faculty of Medicine, University of Southampton, Southampton, UK; 6 University Hospital Southampton NHS Foundation Trust, Southampton, UK

**Keywords:** Complex intervention, Process evaluation, Schizophrenia, Psychosis, Weight management, Logic model

## Abstract

**Background:**

STEPWISE is a theory-informed self-management education programme that was co-produced with service users, healthcare professionals and interventionists to support weight loss for people with schizophrenia. We report the process evaluation to inform understanding about the intervention and its effectiveness in a randomised controlled trial (RCT) that evaluated its efficacy.

**Methods:**

Following the UK Medical Research Council (MRC) Guidelines for developing and evaluating complex interventions, we explored implementation quality. We considered causal mechanisms, unanticipated consequences and contextual factors associated with variation in actual and intended outcomes, and integrated treatment fidelity, using the programme theory and a pipeline logic model.

We followed a modified version of Linnan and Steckler’s framework and single case design. Qualitative data from semi-structured telephone interviews with service-users (*n* = 24), healthcare professionals delivering the intervention (*n* = 20) and interventionists (*n* = 7) were triangulated with quantitative process and RCT outcome data and with observations by interventionists, to examine convergence within logic model components.

**Results:**

Training and course materials were available although lacked co-ordination in some trusts. Healthcare professionals gained knowledge and some contemplated changing their practice to reflect the (facilitative) ‘style’ of delivery. They were often responsible for administrative activities increasing the burden of delivery. Healthcare professionals recognised the need to address antipsychotic-induced weight gain and reported potential value from the intervention (subject to the RCT results). However, some doubted senior management commitment and sustainability post-trial.

Service-users found the intervention highly acceptable, especially being in a group of people with similar experiences. Service-users perceived weight loss and lifestyle benefits; however, session attendance varied with 23% (*n* = 47) attending all group-sessions and 17% (*n* = 36) attending none. Service-users who lost weight wanted closer monitoring and many healthcare professionals wanted to monitor outcomes (e.g. weight) but it was outside the intervention design. No clinical or cost benefit was demonstrated from the intermediate outcomes (RCT) and any changes in RCT outcomes were not due to the intervention.

**Conclusions:**

This process evaluation provides a greater understanding of why STEPWISE was unsuccessful in promoting weight loss during the clinical trial. Further research is required to evaluate whether different levels of contact and objective monitoring can support people with schizophrenia to lose weight.

**Trial registration:**

ISRCTN, ISRCTN19447796. Registered 20 March 2014.

## Background

Obesity and excessive weight gain pose a serious health concern for people with schizophrenia. The prevalence of obesity and rates of cardiovascular disease and type 2 diabetes are 2–3 times higher than in the general population and, weight gain is a key contributor to the excess morbidity and mortality with accelerated rates of obesity contributing to reduced life expectancy of between 10 and 20 years [1–3]. The impact of weight gain is not confined to poor physical health, but also may further add to the experience of distress and stigma [4]. Susceptibility to weight gain for people with schizophrenia can be explained by many factors. As well as the impact of antipsychotic medication on weight gain [5], schizophrenia can affect neuro-endocrine functioning as well as impairing cognition and motivation to modify daily routines. The disease and treatment interact with environmental factors such as poor diet [6–8], social isolation, physical inactivity and poverty [7–10]. Thus, the development of effective weight loss treatments to support people with schizophrenia must overcome challenges posed by a varied set of antecedent factors.

Complex interventions are those with “several interacting components” [11]. The theory-informed STEPWISE programme was developed using the well-established approach to developing type 2 diabetes education - Diabetes Education and Self-Management for Ongoing and Diagnosed (DESMOND) [12]. It involves interactions between healthcare professionals, service users and equipment. The multifaceted nature of STEPWISE can create problems when determining what affected outcomes and the extent to which the intervention could be standardised. Complex interventions tend to be more sensitive to features of the local context, with long and complex causal chains linking the intervention with the outcome. As such, they can be difficult to evaluate and it has been argued that RCTs may not be the best method of evaluation as they seek to remove variation between interventions, agents and contexts and how they interact [13].

We conducted an RCT to assess the effectiveness of STEPWISE. The trial protocol and main results have been published previously [14, 15]. In brief, between 10 March 2015 and 31 March 2016, we recruited 414 people with schizophrenia, schizoaffective disorder or first episode psychosis from ten mental health organisations in England. Participants were randomly allocated to the STEPWISE intervention or treatment as usual. The STEPWISE programme was a 12-month intervention which comprised a foundation course of four weekly 2.5-h sessions delivered by two trained healthcare professionals or associated staff; one-to-one support contact of ~ 10 min every two weeks, usually by telephone, and additional group-based sessions of 2.5 h scheduled at four, seven and 10 months post-randomisation. The intervention is described in full elsewhere [15].

We conducted a process evaluation, in line with MRC guidance alongside the RCT, to assess fidelity and quality of implementation, and sought to clarify causal mechanisms and identify contextual factors associated with variation in outcomes [16].

## Methods

### Theoretical framework

Qualitative research was undertaken alongside the RCT to explore implementation of the intervention. We sought to understand how service users and facilitators responded to the intervention [17–19] and potential causal pathways to success or failure [17–20]. The evaluation’s rationale was principally pragmatic [21] as we sought a basis for ‘organising future observations and experiences’ [22], and investigating plausible real-world consequences [23] of future decisions.

We used a modified version of Linnan and Steckler’s framework [24] and a single case design with the unit of analysis at the service user (*n* = 24) and the intervention programme (*n* = 20 healthcare professionals; *n* = 7 interventionists) levels.

The World Health Organisation (WHO) International Classification of Functioning (ICF) for schizophrenia was used as a conceptual framework for describing ‘context’ [25, 26]. Similar published studies [27, 28] informed a priori the topic guide for service user interviews. Normalisation Process Theory (NPT), a model used to support implementation and evaluation of innovations [29–32], formed the basis of the topic guide for healthcare professional interviews. To characterise stakeholder’s understanding of the intervention we used the Theoretical Domains Framework (TDF), an approach to determine causes of behaviours [33]. TDF codes were mapped post hoc to the theories underpinning the intervention, which were Leventhal’s Self-Regulation Theory [34], Bandura’s Self-Efficacy Theory [35], and Marlatt & Gordon’s Relapse Prevention Model [36].

**Fig. 1:**
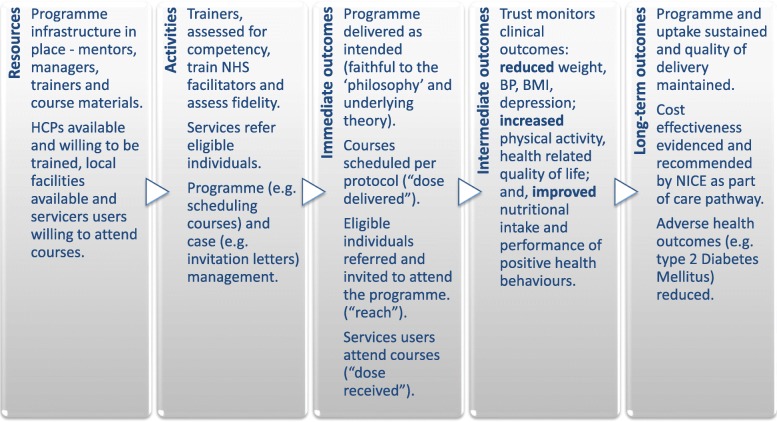
Summary of logic model components for the implementation of the STEPWISE intervention. Legend: *HCP* Health Care Professional, *BP* Blood Pressure, *BMI* Body Mass Index, *NICE* National Institute for Health and Care Excellence

We considered the programme components critical to successful replication of the intervention theory, along with potential causes of failure from these components interacting together [37–39], to develop a logic model [40, 41]. A summary of logical model components and intended outcomes is shown in Fig. 1.

Recruitment, reach, intervention dose delivered/received and fidelity were characterised, both qualitatively and quantitatively, based on the National Institute for Health Behaviour Change Consortium’s (NIHBCC’s) approach to treatment fidelity [42]. We described context qualitatively, and triangulated all data sources, which included interventionist, service user and facilitator interviews, RCT data and quantitative fidelity data.

### Participant selection

Written informed consent was obtained to approach all service users as part of the RCT and re-consent (audio-recorded) took place for those sampled (intervention arm only) prior to the interview. Healthcare professionals were approached and consented via telephone (audio-recorded), and interventionists by email. Written and verbal (audio-recorded) consent was documented using NHS REC-approved written consent forms and were provided to all participants. Service users were purposively sampled by study site, gender and age. A summary of selected characteristics of the service users sampled is shown in Table 1. Facilitators (*n* = 40) were purposively sampled by site, occupation, gender and experience of group facilitation. A summary of education and professional categories of facilitators is shown in Table 2. Seven interventionists (all those involved in developing the intervention) were interviewed.

**Table 1:** Summary of selected characteristics of service users interviewed

Age (years)	n	Sex	n	Ethnicity	n	Dx	n	Outcome (weight)	n	Sessions attended	n
18–25	5	Male	12	White British	19	F20	11	Weight loss (CI)	7	0	0
26–35	7	Female	12	African	2	FEP	8	Weight loss (NCI)	6	1–2	1
36–45	8			White Other	1	F25	5	Weight gain (CI)	3	3–4	4
46–55	4			Bangladeshi	1			Weight gain (NCI)	6	5–6	12
				Indian	1			No data	2	7	7

*FEP* First Episode Psychosis, *F20* Schizophrenia, *F25* Schizoaffective disorder; *CI* Clinically important, *NCI* Not clinically important, *Dx* Diagnosis

**Table 2:** Summary of selected characteristics of healthcare professionals interviewed

Sample Characteristics	n
Education	
Undergraduate degree	10
Postgraduate degree	3
Postgraduate diploma	2
Other	4
No data	1
Professional Category	
Mental Health Nurse	8
Occupational Therapist	3
Support Worker	2
Research staff	2
Physiotherapist	1
Pharmacy Technician	1
Dietician	1
Healthy Living Advisor	1
Community Development	1

### Data collection

Qualitative data were collected via semi-structured telephone interviews with service users (*n* = 24) and facilitators (*n* = 20) by RG-W, using piloted topic guides [16]. Unstructured interviews with interventionists (*n* = 7) were conducted by DH to explore observations during fidelity assessments, elements from the behaviour change wheel [43], the NIHBCC framework [42] and the Programme logic model.

All interviews were audio-recorded using an encrypted device and transcribed verbatim. Table 3 shows median (range) duration of interviews. Data saturation was achieved in all datasets.

**Table 3:** Median (range) duration of interviews (minutes)

Interviewee category	Number interviewed	Median duration (range) in minutes
Service users	24	18:57 (13:06, 30:33)
Facilitators	20	46:13 (29:29–79:32)
Interventionists	7	39:20 (43:39–64:00)

Transcripts or analyses were not returned to interviewees. Field notes captured during and after interviews were reviewed by RG-W, DH, KBK, DS, AE and LS. No prior relationship was established between researcher (RG-W) and service users. RG-W was known to two facilitators (dual role as research nurse) prior to interview. Two interventionists knew DH, from project management meetings, prior to interview.

### Data analysis

Transcripts were coded systematically (RG-W and DH) using QSR International (Warrington, UK) NVivo version 11. Table 4 summarises the systematic and opportunistic coding by interviewee group. A sample of transcripts were reviewed by KBK, DS, AE and emerging themes discussed.

**Table 4:** Summary of coding by interviewee group and source

Interviewee group	Theory/ Constructs					
	TDF domains	NIHBCC framework	NPT	Logic Model	BCT	Acceptability
Service users	Systematic	Opportunistic		Opportunistic	Systematic	Systematic
Facilitators		Opportunistic	Systematic	Opportunistic		Opportunistic
Interventionists		Systematic		Systematic	Systematic	

*S* Systematic, *O* Opportunistic, *BCT* Behaviour Change Taxonomy, *TDF* Theoretical Domains Framework [45], *NPT* Normalisation Process Theory [30–33], *NIHBCC* National Institute for Health Behaviour Change Consortium

### Fidelity assessment

Fidelity assessment of the intervention was assessed via direct observation using the STEPWISE Core Facilitator Behavioural Observation Sheet (CFBOS) which tests for the presence or absence of 35 behaviour domains relating to 9 items assessing behavioural change, planning and goal-setting. The DESMOND Observation Tool (DOT) also measured facilitator versus service user talk time using timed audio cues during a sample of sessions. There is evidence to indicate a link between more effective receipt of self-management education and less facilitator talk [44]. Fidelity strategies are summarised in Table 5. Facilitator attrition was also recorded. A programme like STEPWISE would require national and local infrastructure for quality control and mentoring but was not available during our study; therefore, facilitators did not receive formal feedback nor could evaluation of these components take place.

**Table 5:** Summary of strategies intended to ensure fidelity of the STEPWISE programme

Fidelity Components				
Design	Training	Delivery	Receipt	Enactment
• Theory based intervention with treatment dose (i.e. number, frequency and duration of sessions) specified in the protocol. • Protocol deviations recorded. • Risks to implementation were mitigated by: 1) piloting the programme in one Trust prior to the RCT; 2) setting a minimum for the number (*n* = 4) of facilitators per Trust; and, 3) the tracking of trained staff availability and attrition.	• Written materials and facilitator training were standardised across providers; and, intended delivery style was modelled by expert trainers. • Facilitators used role play to test skills and, reflected on their own performance and skill acquisition and made changes (as required). • Optimum skillset for the role (including one of two having clinical skills) defined for providers. • Level of education and experience of physical and/or mental health and group work captured. • Peer support available during delivery.	• Service user feedback after sessions, semi-structured interview (after foundation course) and facilitator observations informed on the credibility of facilitators, non-specific treatment effects and differences across providers. • Training materials, including resource lists, supported standardisation across providers. • Adherence was monitored via recording attendance, facilitator self-reflection and direct observation of content and delivery; local coordination and monitoring by providers; and, facilitator and service user interviews. • Contamination (of trial arms) was minimised by standardised study design training and on the ground instructions, regular supervision and on-site and remote monitoring.	• Service users invited to participate in sessions (e.g. discuss answers to questions with others); and, facilitators used scripted summaries to aid understanding and check comprehension. • To ensure ability to use cognitive skills (e.g. goal setting and monitoring progress) and perform behaviour skills (e.g. identify and manage triggers), sessions encouraged identification of (and ways to overcome) obstacles; and, per-session (and overall) feedback was invited. Self-monitoring was encouraged and 1:1 support was provided by facilitators.	• Interviews invited feedback on the purpose of the intervention and experiences (skills, behaviours, goals); and explored learning and use (or not) of skills by service users and facilitators (self-report). • Adherence (frequency and duration) of sessions delivered was monitored. • Booster and telephone support maintained for 12 months.
Fidelity goals not monitored (or applicable)				
• Equivalent dose is not applicable as there was no active control.	• No strategies were employed to minimise “drift” in facilitator skills as no benchmark had been established.			

Components and fidelity goals derived by the Behaviour Change Consortium recommendations for enhancing treatment fidelity (BCCr) [49]

### Triangulation

A triangulation protocol [45, 46] was used to compare outcome data from the RCT [47], qualitative and quantitative process data and observations during fidelity observations. The presence and level of convergence was examined within the 18 components of the logic model. We did not prioritise any one data source over the other in assessing the intervention. Feedback on the results of triangulation was integrated into the findings. We did not formally assess the coding between researchers due to time limitations.

## Results

### Context

We used the WHO ICF [25, 26] to explore ‘context’ for people with schizophrenia and considered the challenges which may be experienced, either as a direct result of symptoms and medication, or environmental factors (e.g. stigma) that may have impacted on service users’ interaction with the intervention in the broadest sense.

Whilst some people with schizophrenia are high functioning but experience hallucinations and/or delusions, others are more cognitively challenged requiring consideration of how the information can be delivered and in what ways.

*There could be like mild learning disabilities running alongside their mental illness as well and that, I have definitely noticed that that*’*s hindered* .*.*. *someone*’*s ability to engage.* (Facilitator S09/F03)

**Fig. 2:**
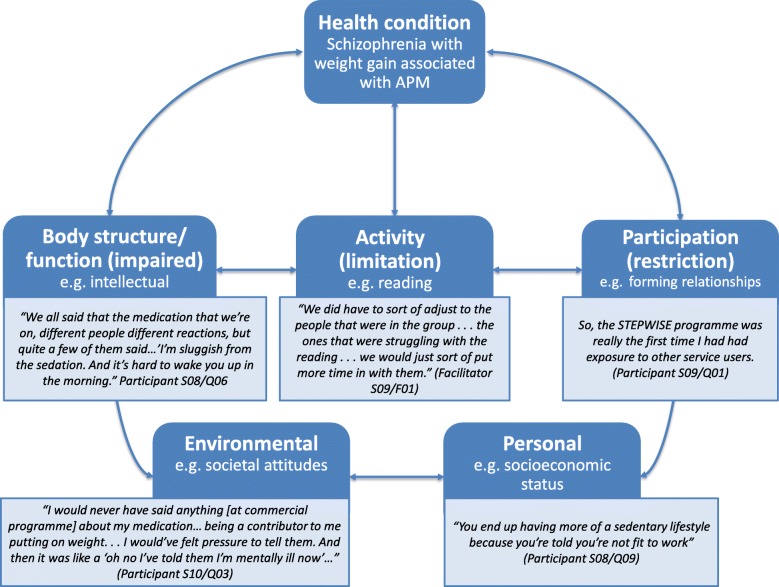
Summary of context for people with schizophrenia informed by the ICF conceptual framework. Legend: *ICF* International Classification of Functioning. Reprinted from Towards a common language for functioning, disability and health ICF, World Health Organization, Diagram (‘model of disability’), Page 9, WHO Reference Number: WHO/EIP/GPE/CAS/01.3, Copyright 2002. APM = Antipsychotic medication

Contextual factors considered in the implementation of the intervention are summarised in Fig. 2.

Of those service users interviewed, three lost and three gained a significant amount of weight. Two of those who gained weight had first episode psychosis and reported more symptoms, whilst almost all service users talked about self-monitoring (e.g. support tools). When ordered by weight loss, only the top three service uers talked about self-belief and persuasion.

When he phoned me... It was like, “how did you know I was eating rubbish?"... But he was really good, like really motivating. It was like, “I'm going to see him in February; I don't want [him] to see I’ve put on what I’d lost.” (Service user S01/Q01, -12.7kg).

“ … if I wasn't in, they [the facilitator] would leave a postcard saying, you know, you can do it. And it was really nice, really kept me going.” (Service user S08/Q05, -9.3kg)

Those who succeeded in losing weight valued monitoring and feedback from others, although monitoring and feedback of behaviour or outcomes was not explicitly part of the intervention and they wanted more monitoring.

“It needs to be longer … like 10 weeks … give us updates on how we are coping, on what we are doing … to monitor us more closely … it was becoming a routine and then it just stopped … ” (Service user S04/Q02, -6.1kg)

“more regular support … see how you’re doing, because … the next session’s quite far away” (Service user S08/Q05, -9.3kg)

### Triangulation

**Fig. 3:**
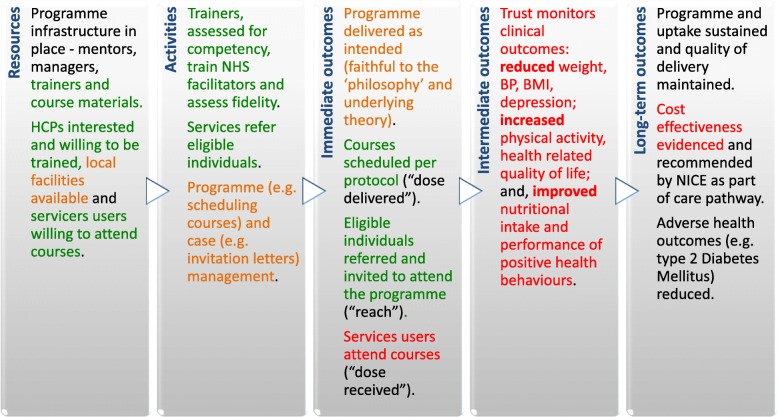
Summary of triangulated findings within logic model components. Legend: Areas are highlighted to indicate where findings from the data triangulation supported (green), diverged (red) or varied (amber) when analysed against the theory of change

We triangulated quantitative and qualitative findings with data types contributing to 14 and 16 (of 18) logic model components. Agreement on three components was found, partial agreement on two, and dissonance on seven components. Six components were found to be silent which was anticipated due to these areas only being amenable to one method of assessment. In some areas of dissonance, we found data were measuring difference elements of the constructs e.g. committed resources as opposed to how the result was valued. Analysis of qualitative data revealed nuances in some areas e.g. inadequate resource allocation for co-ordination. A summary of triangulated findings is presented within components of the Logic Model in Fig. 3.

### Resources

**Fig. 4:**
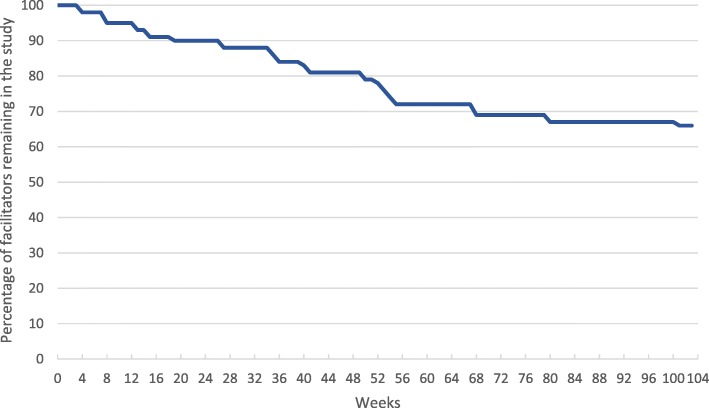
Time (weeks) to facilitator attrition from foundation training

Training resources were found to be adequate according to healthcare professionals. Course materials were universally popular although availability of venues was sometimes limited. Healthcare professionals seemed motivated to become STEPWISE facilitators; and whilst many facilitators continued to support STEPWISE, over the course of 2 years, a third stopped largely because of job moves (Fig. 4).

Healthcare professionals and interventionists interviewed felt buy-in from senior managers in some trusts was insufficient.

“Anything above team leader level... there is no expectation really... they all go yes it's lovely, but... the Trusts and the NHS do not then provide us with the resources to be able to do it.” (Facilitator S05/F02)

“Within this particular trust, one of the deputy directors had been involved right from the start and he was very keen for it to happen so it’s been sort of disseminated downwards from there” (Facilitator S03/F05)

In some trusts, healthcare professionals delivering the intervention felt coordination of resources were not adequate. Service users were motivated to participate (82.6% attended at least 2 of 7 group sessions), liked the course materials and how the sessions were run; however, some healthcare professionals doubted the sustainability of delivery outside the RCT.

“I think the resources were excellent... I think that would be a concern going forward... there wouldn’t be funds... for us to have taxis for everyone and some of the other resources we’ve been given... Where would that money … come from? I think the taxi thing was really helpful. A lot of people probably wouldn’t have come without the taxis.” (Facilitator S06/F04)

### Activities

Facilitator training was delivered as scheduled with additional training provided to NHS trusts where required. Healthcare professionals highlighted that planning and delivery of sessions took significant resources, greater than advertised, and often felt stress as a result of under-resourced programme and case management. Healthcare professionals and interventionists highlighted the importance of co-ordination activities to programme delivery. Clinical gatekeeping, restricting access to eligible services users, was not thought to be widespread.

“It took almost the entire day by the time we’d been out to buy the stuff that we needed for the lunches and set the room up and do the preparation...and then following the group sort of just writing the notes and things up, so it was a day out effectively a week.” (Facilitator S08/F05)

“Had it not been for [the co-ordinator] … it would have took me a full day. It's only because she had everything organised, and ... she knew what we needed for each week...” (Facilitator S01/F02)

“there were sites where you had a very proactive coordinator … in some other places … that wasn’t quite the case … it was very much left to them [facilitator]” (Interventionist, D03)

“Some of the feedback that I was getting during telephone conversations was … people with mental health problems might need something additional … they’ve got mental health problems...other stuff...difficult social stuff...adapting it straight from a study for people with diabetes whether or not they have like co-morbidity... it might not be as transferable.” (Facilitator S01/F04)

### Immediate outcomes

#### Fidelity of intervention delivery

We examined immediate outcomes associated with delivery of the intervention using the Behaviour Change Consortium recommendations for enhancing treatment fidelity (BCCr) [48]. Fidelity assessment showed mean (SD) facilitator talk time was 47.6% (12.2%) and facilitator behaviours deemed positive was 54.1% (17.6) with a range between centres of 31.8% (13.2%) to 64.6% (17.67%). Lapses in fidelity were observed by assessors e.g. giving answers, rather than eliciting, solutions from service users to discussion topics.

All foundation and all but one booster sessions were delivered as scheduled. A total of 171 (82.6%) service users attended at least 2 (of 7) group sessions. Approximately 23% (*n* = 47) of service users attended all sessions; however, 17.4% (*n* = 36) did not attend any sessions.

Whilst the trial included 414 people with schizophrenia, schizoaffective disorder or first episode psychosis and less than 20% dropped out (‘*reach*’), this number represents only a small proportion of those potentially eligible at study sites.

#### Service user acceptability

Using Sekhon’s acceptability framework [31], we found service users were extremely positive about the intervention. Most respondents found the provision of taxis, lunch and support tools for each session highly acceptable. They understood the purpose of the intervention and found it realistic, flexible, accessible and not burdensome. Service users perceived benefits to include lifestyle change and/or weight loss although this was not always the case.

“Oh yes definitely, I've lost quite a bit of weight and I'm in much better shape … ” (Service User S06/Q06; -12.3 Kg at 3 months, lost to follow-up at 12 months)

“ … it helped me lose weight and it helped me write down what I was eating, what I was drinking, and helped me do more exercise.” (Service User S04/Q08; 29.7 Kg 0-12 months)

There was an overwhelming sense that service users found the peer group interaction and social support to be beneficial. Some service users had never met others with schizophrenia and had attempted weight loss at commercial programmes but did not want to talk about the impact of their medication because of stigma attached to severe mental illness.

“I find it fantastic … meeting other groups of people on a similar medication with similar problems...” (Service User S01/Q01)

Through triangulation of service user perspectives, RCT outcomes and analyses of intervention components we found that most service users talk about self-monitoring (support tools); more successful service users seem to value monitoring and feedback by others; however, objective monitoring and feedback of behaviour and outcomes by others was not part of the intervention design.

#### Healthcare professionals (facilitators)

**Fig. 5:**
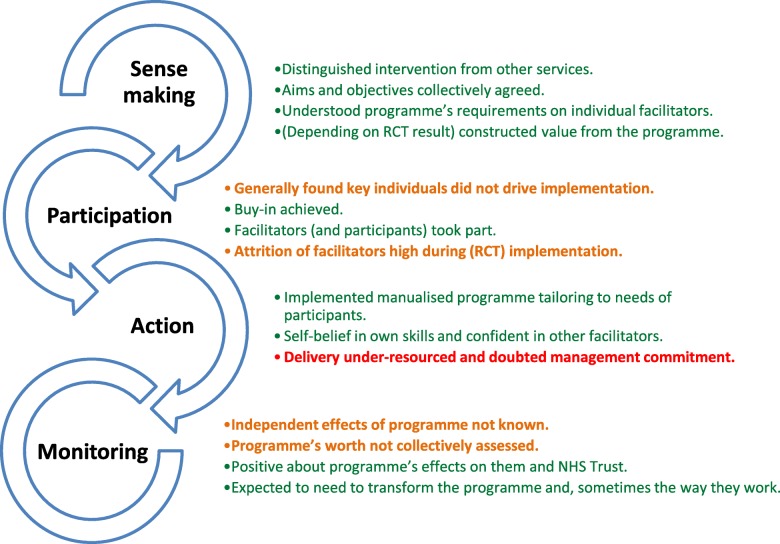
Summary of the facilitator qualitative findings understood through the Normalisation Process Theory. Legend: May CR. BMC Health Serv Res. 2011;11(1):245

A summary of qualitative findings understood through the Normalisation Process Theory [32] is shown in Fig. 5. Most healthcare professionals distinguished STEPWISE from other interventions, reported understanding what was required of them, shared its aims with other healthcare professionals*,* believed it was the right thing to be doing and constructed potential value from the intervention.

“I think that it’s definitely something that we haven't been doing. And I think it's something that we should be doing.” (Facilitator S02/F06)

Healthcare professionals reported that service users’ understanding of the intentions of the programme was not immediately or always understood. Some felt this was due to cognitive difficulties or services users being more familiar with a didactic, rather than, facilitative approach.

“And their functioning is quite different, you know, there's really quite a high level functioning lady there who sort of understood the thing straight away, and another guy … it's very difficult to find out what his understanding actually is. Because he tends to talk in slightly psychotic terms.” (Facilitator S05/F02)

“We do repeat, you know … it's a lifestyle choice... we're not saying that you've got to ban all bad food... they think they're going to go there, get weighed, and they'll go on a diet, we're going to give them a diet sheet. And for some people that would work, and for other people empowering them works... sometimes the like peer pressure and going to Weight Watchers and knowing that you know there's some expectation you've lost some weight, because it's been marked down, that works very well for some people but not others.” (Facilitator S01/F02)

Healthcare professionals could perform the tasks required by the intervention and universally complementary about the training and printed materials they received, although one noted:

“It was a lot to take in just those three days initially … quite intense.” (Facilitator S08/03)

Staff highlighted organisational challenges in some trusts including: key individuals not driving implementation, lack of commitment from senior leadership, under-resourced delivery and doubt regarding sustainability of the programme post-RCT.

“If it works … it's going to become part of like the working practice for everybody. We won't be giving out free gifts … It will just be … part of people's care plan.” (Facilitator S01/F02)

The extent to which healthcare professionals could access information about the intervention effects (e.g. weight) was limited. STEPWISE has no mechanisms by which healthcare professionals could access objective information about the effects of STEPWISE.

“No one seems to be taking weight! I'm not gathering data on individual clients … we don't even have an ongoing way of monitoring weight now [in the trust] … ” (Facilitator S05/F02)

Similarly, healthcare professionals reported finding it difficult to ensure services uses comprehended the course content and had sufficient cognitive skills to perform the necessary behaviour change. This was echoed by some service users who wanted more monitoring by others. Interventionists reported that (subjective) information about the extent to which service users understood the material could have been elicited by facilitators during sessions.

“Sometimes it wasn’t explored to check understanding... I would want to say with somebody like summarise what key message you’ve taken away from that session... I don’t think we ... cover it in [facilitator] training... it’s something that could be emphasised more to check people’s understanding.” (Interventionist, D07)

#### Intermediate outcomes

Most service users found the intervention to be highly acceptable and many of those interviewed reported making important changes in levels of physical activity, improved nutrition and reduction in weight; however, RCT outcome data showed that this was only true for a small number of service users. Any differences between intervention and control groups could not be explained by receipt of the intervention.

## Discussion

This process evaluation explored how context affected programme implementation and may help understand why the STEPWISE intervention was unsuccessful in supporting weight loss in people with schizophrenia, schizoaffective disorder and first episode psychosis. The process evaluation found that the programme design required closer (objective) monitoring of service users’ progress towards their lifestyle goals, greater integration of the programme within the organisation, i.e. coordination of resources, supporting staff appropriately to run the programme, and consideration of the context and variation in symptoms for people living with schizophrenia to achieve the desired lifestyle change.

The evaluation identified poverty, under-resourced services and stigma associated with severe mental illness as important issues for providers of tailored weight management programmes, as have others [49]. Service users valued the provision of (funded) transport and found patient-focused educational approach acceptable; however, healthcare professionals felt the level of resource employed would be unsustainable outside of the trial. If born out, ensuring adequate resources would be essential for successful implementation. Recent research conducted by McGinty and colleagues identified ways to adapt and scale up a severe mental illness weight loss intervention (ACHIEVE); including, building staff capacity, engaging leaders and organisational change and financial policy strategies [50] which may overcome some of the perceived and actual resourcing and leadership challenges we found in our study.

We investigated intervention fidelity via direct observation of STEPWISE courses, which is considered the gold standard approach [48]. Observations were undertaken by interventionists; however, qualitative and quantitative process and implementation data were collected and analysed by evaluators (DH and RGW) with sufficient independence to critically observe stakeholders, as recommended in guidance [20].

### Limitations

We coded participant transcripts to the Behaviour Change Wheel [43] during analysis ‘after the event’, rather than integrating constructs within the interview schedule. Therefore, the subsequent analysis did not draw methodologically from these constructs rather it reflected service users’ patterns of attention. Programme infrastructure that would, if rolled out, be required to support training, provision of materials and ongoing quality assurance, was not in place and therefore could not be evaluated. In addition, in at least one trust facilitators were managed by the Research and Development department which meant greater control over resource management for intervention delivery.

### Implications

**Fig. 6:**
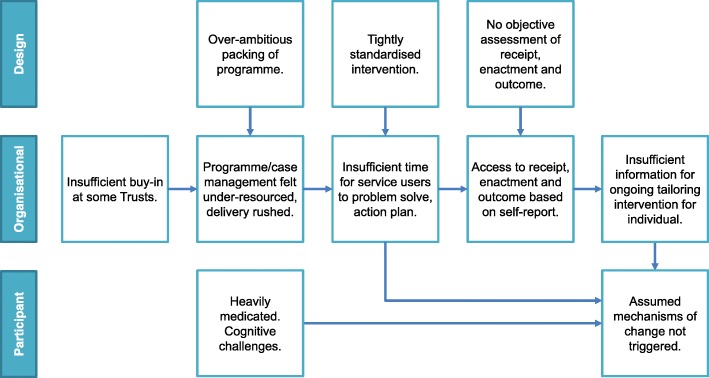
Potential modifications to improve the STEPWISE intervention

As the theory underpinning the intervention is sound, we have explored the intervention design and considered how it has been packaged (session content/timings), along with organisation issues (resources, lack of monitoring/tailoring for individuals) and the context of living with a severe mental illness to assist in understanding why the required behaviour change mechanisms have not been triggered. The process evaluation points to potential modifications which are highly likely to improve the design and organisation of this or similar interventions for this population (Fig. 6).

## Conclusions

The triangulation of qualitative and quantitative findings of the STEPWISE intervention reveal barriers and facilitators that influenced programme delivery. System level infrastructure, local leadership and providing sufficient time and resources for adequate coordination and delivery are essential. There is demand from people with schizophrenia for this type of programme; and, the need for interventions to support weight loss is unlikely to reduce because few tailored programmes exist, and obesity rates generally are rising. Findings from our study suggest certain strategies may improve and support delivery in any future adaptions and in subsequent real-world implementation. Although further research is required to identify what type and format of interventions will best support people with schizophrenia to lose weight, and associated costs, our study found that close objective monitoring of desired outcomes to assess progress towards individual lifestyle goals (e.g. weight) and an adequately resourced programme at local (i.e. personnel, support tools, venues) and national (manual, training, quality assurance) levels are essential. Furthermore, consideration of the range of contextual factors, which vary in their applicability to, and impact on, people living with schizophrenia, will help ensure interventions are tailored and flexible; and therefore, more likely to achieve lifestyle changes which can reduce the health inequalities experienced by services users when compared to the general population.

## Data Availability

The datasets generated and/or analysed during the current study are not publicly available due to ensuring the confidentiality and anonymity of the participants but are available from ctru@sheffield.ac.uk on reasonable request.

## Abbreviations

BCCr: Behaviour Change Consortium recommendations
BCT: Behaviour Change Taxonomy
CTRU: Clinical Trials Research Unit
CFBOS: Core Facilitator Behavioural Observation Sheet
DOT: DESMOND Observation Tool
DESMOND: Diabetes Education and Self-Management for Ongoing and Diagnosed
ICF: International Classification of Functioning
MA: Master of Arts
MRC: Medical Research Council
NIHBCC: National Institute for Health Behaviour Change Consortium
NPT: Normalisation Process Theory
PI: Principal Investigator
PhD: Philosophiae doctor
RCT: Randomised Controlled Trial
SMI: Severe Mental Illness
SD: Standard Deviation
STEPWISE: STructured lifestyle Education for People WIth SchizophrEnia
TDF: Theoretical Domains Framework
WHO: World Health Organisation
